# Tumor histologic grade is the most important prognostic factor in patients with penile cancer and clinically negative lymph nodes not submitted to regional lymphadenectomy

**DOI:** 10.1590/S1677-5538.IBJU.2015.0416

**Published:** 2016

**Authors:** Giuliano Amorim Aita, Stênio de Cássio Zequi, Walter Henriques da Costa, Gustavo Cardoso Guimarães, Fernando Augusto Soares, Thais Safranov Giuliangelis

**Affiliations:** 1 Departamento de Urologia, Hospital Universitário - Universidade Federal do Piauí, Brasil; 2 Serviço de Urologia, Departamento de Cirurgia Pélvica, A C Camargo Cancer Center, SP, Brasil; 3 Departamento de Patologia, AC Camargo Cancer Center, SP, Brasil

**Keywords:** Penile Neoplasms, Prognosis, Penis

## Abstract

**Introduction:**

The presence and extension of inguinal lymph node metastasis are the main prognostic factors in patients with penile cancer. Physical exam and image exams are not adequate to evaluate inguinal lymph nodes and many patients are submitted to non-therapeutic lymphadenectomies. However, it is known that not all patients with clinically or histologically negative inguinal lymph nodes evolve favorably.

**Casuistic and Methods:**

the authors evaluated the clinical and pathologic characteristics of 163 patients with penile carcinoma and clinically negative inguinal lymph nodes followed for three or more years and their impact on global survival (GS) and cancer-specific survival (CSS) in the 10-year follow-up. Primary pathologic tumor stage (p=0.025) and the presence of high grade of tumor differentiation (p=0.018) were predictive of CSS. The presence of high grade tumor was an independent specific prognostic factor of death risk (RR 14.08; p=0.019).

**Conclusion:**

high histologic grade was an independent predictive factor of specific death risk in patients with penile carcinoma and clinically negative lymph nodes followed for three or more years.

## INTRODUCTION

Penile carcinoma is rare in developed countries, and is more frequent in under developed countries. India presents 3.32 cases for every 100.000 inhabitants (1). In Brazil, it accounts for 2% of all tumors in men, reaching up to 10% in some regions (North and Northeast), and it is one of the nations with the highest incidence of this disease (2).

Penile squamous carcinoma (PSC) responds to 95% of all primary malignant tumors and the first site of dissemination corresponds to inguinal lymph nodes. Usually pelvic lymph nodes are only involved when previously inguinal lymph nodes have been affected. Hematogenic dissemination is rare and is observed in less than 10% of patients (3). Without adequate treatment, patients die in two years following diagnosis due to complications related to local growth or metastasis (4).

Presence and extension of involvement of inguinal lymph nodes are the most important prognostic factors related to survival of patients with penile cancer (5). Lymphadenectomy is the only curative treatment, even for patients with lymph node metastasis (3, 4). However, the procedure presents high morbidity, affecting significantly quality of life of patients. Inguinal lymphadenectomy is considered therapeutic for patients with lymph node metastasis and prophylactic for those with primary tumor with at least one risk factor for lymph node involvement: stage ≥T1b and/or palpable lymph nodes following treatment with antibiotics and/or unknown follow-up.

Patients without risk factors and who are committed to treatment theoretically comprise the low risk group for inguinal metastasis and are spared from prophylactic lymphadenectomy, assuming that lymph nodes are not affected. Also, some patients are not submitted to lymphadenectomy due to different reasons.

Although it is suggested that some patients with clinically negative lymph nodes may have a better evolution than those with positive inguinal lymph nodes, the absence of lymph node metastasis is not equal to therapeutic success. Some of them may present local and regional recurrence, tumor progression and death due to cancer. We evaluated some prognostic factors for global survival (GS) and cancer-specific survival (CSS) in a historical series of patients not submitted to lymphadenectomy and that did not show lymph node metastasis in a minimum follow-up of three years. The occurrence of lymph node metastasis following that period is extremely rare (6–8).

## MATERIAL AND METHODS

In order to perform the study, 163 patients with penile carcinoma and clinically negative lymph nodes followed-up for three or more years, from a data bank of 279 patients were selected. Patients were treated at the Department of Pelvic Surgery of Hospital A.C. Camargo (São Paulo SP) from 1953 to 2012. Patients with incomplete clinical or pathological data, without adequate paraffin block available for pathological analysis, submitted to adjuvant chemo or radiotherapy, with non-epithelial carcinoma of penis and those with life expectancy due to co-morbidities inferior to 6 months were excluded.

Pathologic material of all 163 patients were reviewed by specialized pathologists (Isabela Werneck, Fernando Soares). Histologic classification was made according to subgroups described by WHO (9–11), that include twelve different histologic subtypes of penile squamous carcinoma, including the usual squamous carcinoma, basaloid, condylomatous, verrucous, papillary, sarcomatoid, adenosquamous, pseudohyperplasic, cuniculatum, pseudoglandular, condylomatous - basaloid and mixed carcinomas. Each subtype presents different morphologic and pathologic characteristics (11).

Tumors were graded according to differentiation: low grade, intermediate and high grade, using the criteria described by Velazquez et al. (2008) (12). Accordingly, tumor well differentiated were those who presented similarity to normal or hyperplasic squamous cells, different only in relation to the presence of minimal basal or parabasal atypia (Figure-1). Tumors were classified as high grade when there was presence of any grade of anaplasia. After optical magnification, those areas present low or no keratinization, elevated cytoplasm-nucleus relation, thickening of basal membrane, nuclear pleomorphism, aggregated chromatin, prominent nucleolus and numerous mitosis (Figure-2). Tumors were considered moderately differentiated when they had no criteria to be considered low or high differentiated (Figure-3) (12).

**Figure - 1:**
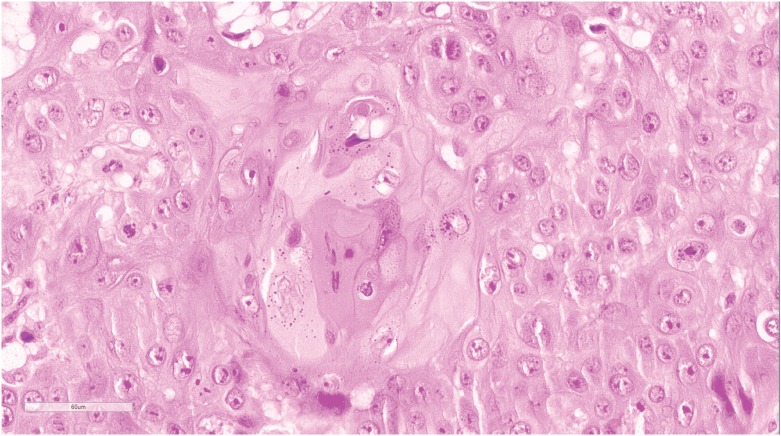
Squamous cell carcinoma of low grade penile. Identifies minimum basal atypia or parabasal. Hematoxylin and eosin. 200x magnification.

**Figure - 2:**
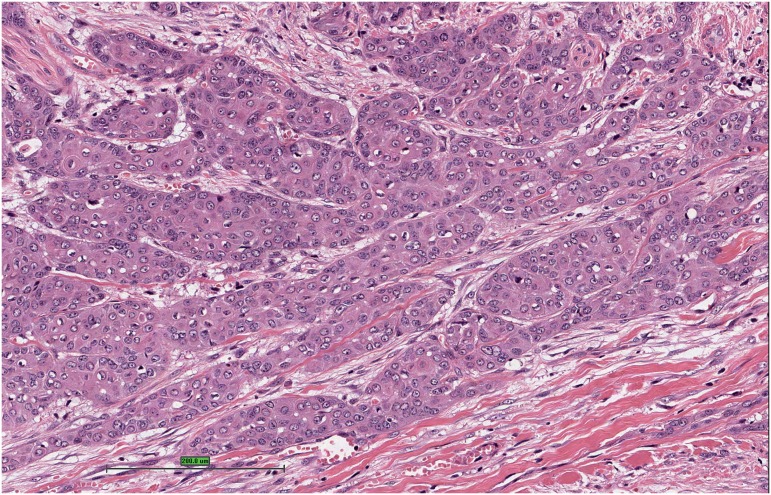
Squamous cell carcinoma moderately differentiated penile. Hematoxylin and eosin.

**Figure - 3:**
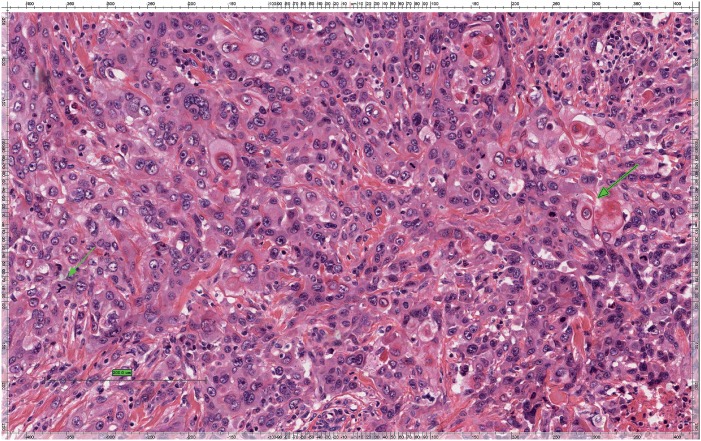
Squamous cell carcinoma high grade penile. dense nuclear membrane, nuclear pleomorphism, aggregated chromatin, prominent nucleoli and numerous mitoses. Hematoxylin and eosin.

The considered variables for each patient were: age, staging according to TNM AJCC 7ed, histologic subtype, tumor differentiation grade, microscopic vascular invasion (MVI), perineural invasion (PNI) and pushing or infiltrating pattern of invasion (microscopic pattern of tumor edges) (13–16). In order to perform the specific study of histologic influence as predictive factor of survival, 12 patients from the initial 163 were excluded, since 5 were considered “evaluation not possible”, and 7 “ignored”, and 151 were considered for statistical analysis (Table 1 and Figure-2). Additionally, it was evaluated follow-up time and clinical situation at the end of the study.

**Table 1:** Clinical and pathologic variables of 163 patients with penile carcinoma with clinically negative lymph nodes - Hospital A.C. Camargo.

Variable	N (%) SD	CSS (%)	P	GS (%)	P
Age (mean)	57 (±12.9)	–	–	–	–
**Stage cN(AJCC7^th^ Ed.)**					
cNO	98 (59.4)	98	0.216	64.3	0.174
cN1	19(11.5)	100		52.6	
cN2	46 (27.9)	93.5		54.3	
**Stage pT(AJCC7^th^ Ed.)**					
pT1a	32(21.2)	100		71.9	
pT1b	10(6.6)	80		70	
PT2	81 (53.6)	97.5		58	
pT3	28(18.5)	96.6	0.025	48.3	0.243
**Histologic grade**					
Low	66 (43.7)	100		63.6	
Intermediate	39 (25.8)	97.4		64.1	
High	46 (30.5)	91.3	0.018	52.2	0.078
**Vascular Invasion**					
Present	15(11.2)	100		66.7	
Absent	119(88.8)	96.6	0.526	57.1	0.908
**Perineural invasion**					
Present	6 (4.5)	100		83.3	
Absent	128(95.5)	96.9	0.698	57	0.47
**Invasion pattern**					
Pushing	50 (35.2)	96.4		56	
Infiltrating	92 (64.8)	96	0.855	59.8	0.947
**Histology**					
Usual SCC	109(66.9)	95.4		60.6	
Warty carcinoma	8 (4.9)	100		25	
Papillary carcinoma	9 (5.5)	100		88.9	
Sarcomatoid carcinoma	3(1.8)	100		0	
Pseudoglandular carcinoma	7 (4.3)	100		57.1	
Carcinoma cuniculatum	19(11.7)	100		63.2	
Verrucous carcinoma	1 (0.6)	100		100	
Other carcinomas	7 (4.3)	100	0.995	100	0.035
Total	163	96.9		60.1	

IBM software Statistical Package for Social Science (SPSS) version 21 was used for statistical analysis. Distribution of clinical and pathologic variables was presented as contingence tables.

Identification of independent factors related to GS and CSS was performed by multiple logistic regression. Selected variables for multiple study were chosen among those who reached statistical significance at univariate analysis, as well as those who presented p values of up to 0.20. Final result of multivariate models, logistic regression and COX (1972) were obtained by stepwise forward selection: from the highest significant variable, it was added one by one every variable in an ascending order.

In order to analyze GS and CSS it was used the Kaplan and Meier estimator (1958) in order to estimate the probability of a patient with penile carcinoma to be alive or not until the time point considered. In order to compare the estimated curves for each category of a determined variable it was used the LogRank non-parametric test. Significance level was 5%.

## RESULTS

Table-1 shows the distribution of studied patients according to clinical and pathologic criteria and the impact on GS and CSS. Medium and mean follow-up were 131 and 150 months, respectively.

In relation to tumor differentiation grade, there were 66 patients with low grade tumor (43.3%), intermediate grade in 39 (25.7%) and high grade in 46 (30.9%). The most prevalent histologic type was usual squamous cell carcinoma (66.9%).

MVI and PVI were present in 11.2% and 4.5% of patients, respectively. Tumor invasion was infiltrating in 64.8% of tumors and pushing in 35.2%. There were 67 deaths (40.6%), 6 due to cancer (6.3%).

GS and CSS in 10 years of follow-up were 97.9% and 60.1% respectively. Among studied parameters, pathologic stage of primary tumor (Pt) and histologic grade influenced CSS at univariate analysis (Figure-2). Pt stage and histologic grade were selected for multivariate analysis. For that purpose, patients with intermediate grade were analyzed together with those with high grade. High grade was an independent predictive factor for GS and CSS. Patients with high grade tumor had higher probability of death due to cancer (RR 14.8; p=0.019) and global deaths (RR 1.86; p=0.023) when compared to those with low or intermediate grade (Table-2).

**Table 2:** Cox regression. Analysis of GS and CS.

Variable	CSS			GS		
	RR	95% CI	P	RR	95% CI	P
**pT stage**						
pT3 vs. pT1 - pT2	1.09	0.12 – 10.10	0.917	1.68	0.91 – 3.08	0.095
**Histologic grade**						
High vs intermediate-low	14.08	1.55 – 25.05	0.019	1.86	1.09 – 3.19	0.023

## DISCUSSION

Squamous carcinoma of penis is a loco-regional disease with a presumable pattern of dissemination, mainly through lymphatic spread. Sequentially, it affects inguinal and pelvic lymph nodes. Presence and extension of lymph nodes metastasis determine the evolution and survival of patients, overlapping any clinical or pathologic criteria of primary tumor (19). Clinical exam fails to predict lymph node metastasis. 20% of patients with negative physical exam show micro-metastasis when submitted to inguinal lymphadenectomy. Available image exams are inaccurate for evaluation of lymph nodes with significant under-staging or over-staging (20). However, absence of lymph node metastasis is not a guarantee of therapeutic success. Factors related to primary tumor may determine different evolutions in this group of patients. Patients who present at least one risk factor for lymph node metastasis are submitted to inguinal lymphadenectomy. On the other hand, patients without any risk factors are spared of the intervention.

However, in this group of patients, some (3.1%) died due to cancer. Very few series analyze exclusively patients not submitted to inguinal lymphadenectomy. As expected, these patients not submitted to inguinal lymphadenectomy present higher rate of CSS (96.9%) and GS (60.1%) than those submitted to inguinal surgery in our institution, including those with pathologic negative lymph nodes (87.1% and 52.7% respectively) (16) and those with positive lymph nodes (64.1% and no data presented, respectively) (21).

Since this was an historic series, several patients presented risk factors of inguinal metastasis: 78.7% with stage > T1a; 30.5% of high grade tumors and stages cN1 and cN2 in 39.4% but were not submitted to surgery for several reasons. It is important to have in mind that the indication of inguinal lymphadenectomy depends on the surgeon, and that referral suffered many changes in our institution throughout those six decades. In spite of that, in this group, classic prognostic parameters described such as angio-lymphatic embolization and perineural invasion were not relevant for the clinical outcome in the studied period. Histologic grade was an independent predictive factor of risk of death and death-specific. Although these high risk patients not operated have been included in the studied series, curiously CSS was very high (96.9%). Nowadays, it is not correct to not operate these patients, in view of the accumulated knowledge and several published guidelines that recommend inguinal lymphadenectomy. Still, those patients did not show inguinal recurrence following 3 years of follow-up, confirming that those were lymph node-negative. Which would be the justification for this disparity (good evolution vs adverse factors)? Maybe the explanation involves something intangible or highly subjective or the pointed clinical judgement of the physicians that decided not to perform the inguinal surgery.

Several studies confirm the negative predictive effect of histologic high grade of penile car carcinoma. A retrospective review of American SEER database involving 593 patients with penile cancer and cNo stage recognized the high grade of differentiation as an independent predictive factor of death risk (RR 3.22; CI 95% (2.0-5.3)) (22). A German group (23) analyzed the role of p16INK4a expression as prognostic factor of penile carcinoma and additionally identified in a multivariate analysis the histologic grade (p=0.049; RR 2.47; CI 95% [1.00-6.09]) as an independent predictive factor of death due to cancer in 5 years. A Brazilian study followed up 648 patients for a median period of 11 months and observed higher CSS in 10 years among those with well differentiated tumors when compared to those with moderate differentiation or undifferentiated regardless surgical specific treatment (log rank p<0.0001 and p=0.006 respectively) (19). Different rates of 5 year-GS were observed according to tumor differentiation grade in a series of 89 patients with a median follow-up of 23 months (24). Patients with high grade tumor had lower GS in 5 years than those with moderately differentiation or low grade (53% and 29% respectively; p=0.01). At multivariate analysis, this finding was not confirmed.

Also, European Association of Urology highlight the importance of tumor differentiation grade of penile cancer. Histologic grade and primary tumor stage are the most relevant criteria for the selection of patients for inguinal lymphadenectomy (8).

The proportion of low and moderate grades has already been presented in previous studies as of minor relevance than the relative presence of high grade tumor, invariably associated with worse prognosis (25, 26). However, it is known that penile tumors are frequently heterogeneous and may exhibit more than one grade, making histologic evaluation difficult. Also, the absence of standardized morphologic criteria for classification in different grades contributes to low reproducibility among pathologists. Graduation in differentiation extremes as performed by our group (grade 1 with very little differences in relation to normal squamous epithelium and grade 3 for tumors comprising anaplasic cells) makes classification easier and provides more consistent prognostic information. Grade 2, more susceptible to subjective interpretation depending on the pathologist, corresponds to all cases not classified as 1 or 3 (27).

In our study, pathologic evaluation of tumor was made only with hematoxylin-eosin staining. More studies are needed to validate and reproduce this high important prognostic criteria in penile carcinoma. The value of additional immune-histochemical studies for evaluation of histologic grade may be addressed by future researches.

## CONCLUSIONS

In this series of patients not submitted to inguinal lymphadenectomy and that did not regionally progress after three years, a small subgroup of patients died due to cancer. Main independent prognostic factor for CSS was the presence of high grade primary tumor. Patients not operated but with high grade tumors that refuse surgery comprise a high risk group and require a more diligent follow-up.

High histologic grade remains a risk factor for death due to penile carcinoma, even in subgroups without lymph node metastasis.

## Abbreviations

SCP: = squamous carcinoma of penis
Ci: = confidence interval
MVI: = microscopic vascular invasion
PVI: = perineural invasion
RR: = relative risk
CSS: = cancer specific survival
GS: = global survival
